# Tomographic findings in Meckel’s diverticulitis

**DOI:** 10.1590/0100-3984.2017.0215

**Published:** 2019

**Authors:** Rômulo Florêncio Tristão Santos, Tiago Kojun Tibana, Carlos Fernando Rio Lima Filho, Edson Marchiori, Thiago Franchi Nunes

**Affiliations:** 1 Universidade Federal de Mato Grosso do Sul (UFMS), Campo Grande, MS, Brazil.; 2 Universidade Federal do Rio de Janeiro (UFRJ), Rio de Janeiro, RJ, Brazil.

**Figure 1:**
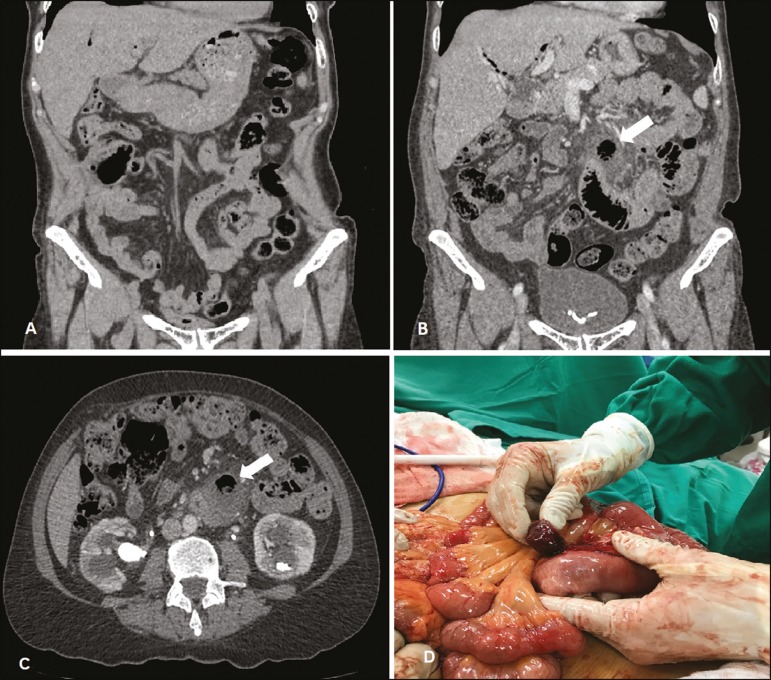
Coronal CT (**A**), acquired six months prior, in which no diverticulum is visible. Coronal and axial CT scans (**B** and **C**, respectively), obtained at admission, showing a diverticulum with signs of adjacent inflammation (arrow). **D:** Laparotomy corroborating the radiological findings and confirming the diagnosis of Meckel’s diverticulitis.

Dear Editor,

A 53-year-old woman presented with a two-day history of sudden-onset epigastric pain that radiated to the right hypochondrium and worsened progressively, together with nausea and chills. She reported no other signs or symptoms. The patient had undergone cholecystectomy 20 years prior and revision of the biliary tract 4 years prior. She was also being followed as an outpatient for nephrolithiasis. Physical examination (palpation) revealed abdominal pain in the right hypochondrium and in the periumbilical region, and there were no signs of peritonitis. Laboratory tests showed no significant alterations. In the radiology report for a computed tomography (CT) scan performed 6 months prior (Figure 1A), there was no mention of a diverticulum. An abdominal CT scan obtained at admission showed a diverticulum at the mesenteric border of the mid-ileum, with signs of adjacent inflammation, characterized by fat densification, without evidence of pneumoperitoneum or intestinal obstruction (Figures 1B and 1C). The patient was submitted to laparotomy, which confirmed a diagnosis of Meckel’s diverticulitis (Figure 1D).

Gastrointestinal malformations account for approximately 6% of all malformations. Meckel’s diverticulum (MD) is the most common such malformation, with a prevalence of 1-4% in the general population. It is composed of all layers of the intestinal wall, characterizing a true intestinal diverticulum. It occurs when the omphalomesenteric duct (yolk duct) fails to obliterate during the first trimester of fetal life^(^^1^^)^. Most individuals are asymptomatic and are typically diagnosed when they present complications such as ulcerations, gastrointestinal bleeding, bowel obstruction, intussusception, diverticulitis, perforations, and neoplasms. MD can also be diagnosed incidentally during laparoscopy or laparotomy performed for other reasons^(^^2^^,^^3^^)^. Acute diverticulitis (sudden inflammation of the diverticulum), as occurred in the case reported here, is seen in 13-31% of complicated cases of MD, the incidence being highest in the fourth and fifth decades of life^(^^4^^)^.

Because it is difficult to diagnose, MD continues to be a major challenge in medical practice^(^^4^^)^. Various conditions can mimic MD and its complications, such conditions including appendicitis, diverticula in other intestinal segments, ureterolithiasis, intussusception, duplication cysts, angiodysplasia, and hemorrhagic tumors^(^^5^^-^^9^^)^. Taken together with the clinical manifestations, the results of some imaging examinations, such as ultrasound and CT, aid in making the correct diagnosis. Because MD without signs of inflammation has a characteristic presentation (a cystic structure with walls structured like those of the intestine) and can cause peristalsis, it can be detected by ultrasound. The inflammatory process increases the vascularization within the structure and can therefore be identified on color Doppler ultrasound^(^^10^^)^. On CT, uncomplicated MD is difficult to distinguish from the normal small intestine. However, a structure with a blind ending, containing gas or liquid, can be observed in continuity with the small intestine. Enteroliths, intussusception, diverticulitis, and signs of intestinal obstruction can also be seen^(^^11^^)^. In cases of inflammation of the diverticulum (Meckel’s diverticulitis), CT can reveal wall thickening with contrast enhancement, fat densification, adjacent fluid collections, or free fluid^(^^3^^)^.

The definitive treatment for MD is surgery, which is always indicated in symptomatic patients. The approach can be by laparoscopy or laparotomy, which provide equally satisfactory results^(^^12^^)^.

The data presented here underscore the importance of diagnostic suspicion for the identification of Meckel’s diverticulitis. In patients with nonspecific abdominal symptoms, radiologists must be familiar with the imaging aspects of MD in order to interpret the imaging studies correctly.
